# Asthma

**Published:** 1910-07

**Authors:** William Francis Waugh

**Affiliations:** Dean of Bennett Medical College, Chicago, Ill.


For Texas Medical Journal.

### Asthma.

BY WILLIAM FRANCIS WAUGH, A. M., M. D., DEAN OF BENNETT MEDICAL COLLEGE, CHICAGO, ILL.

The last word will never be said on asthma. Of all diseases to which humanity is liable there is none in which every case has a more distinct individuality, necessitating specific study. It is a feminine malady—there is no such thing as an ordinary type of woman or of asthma.

Each new revelation following specific study results in the establishment of another type. Formerly we looked on the affection as essentially spasmodic, consisting in a vasomotor spasm in the pulmonary tract. Later it was found that certain cases exhibited vasomotor paresis instead. The importance of the latter feature has been emphasized by Lerch, who finds by the use of the X-ray that many cases have enlargement of a persistent thymus.

These observations show why there have been such contradictory results following various theoretically founded systems of treatment. Looking on the malady as essentially spasmodic, it followed that the relaxants should be employed; and every one of this class has been vaunted. Every one has succeeded, and has likewise failed. Success only follows mathematically when the factors of each case have been recognized and the appropriate remedies administered in effective doses. While lobelin has afforded relief when the spasmodic element is present, strychnine pushed to full dosage has been required when the condition is vasomotor relaxation.

Important as are the vasomotor factors, they are not the whole question. In many cases fecal autotoxemia has been detected, and so important is this feature that it has been asserted positively that the one climate curable for each asthmatic is that in which he or she will take the exercise, etc., that best keeps the bowels regular.

Yet more—when we find an asthmatic who should be eliminating 1200 grains of urinary solids each twenty-four hours is only excreting by this route 300 grains per diem, we can not but surmise that this has something to do with the asthma. The retention of 600 or 900 grains daily of these intensely toxic substances, as shown by examinations of the urine repeated many times, over an extended period, can scarcely fail to be of importance.

A number of clinicians have reported favorably on the use of strychnine arsenate in the paretic forms. This potent remedy was administered in doses of a milligram every one to four hours, and gradually increased as circumstances indicated, until in some cases the daily dose reached one grain, or even two or three grains. These doses were justified by the improvement manifested, this lessening and being re-established by increasing doses; in fact, by the true scientific system of dosage to effect, or "dose enough."

A remarkable clinical observation is the relief following the use of glonoin in both classes of vasomotor disorder, spastic and paretic. Given in doses of a quarter-milligram every ten minutes till the face flushes, this powerful remedy proves almost invariably effective. If there is a state of vasomotor spasm in the pulmonary area, it is relaxed; if the condition there is one of paresis with passive engorgement, the general circulation is opened up and the surplus blood drains away from the lungs. In either case relief ensues. Glonoin is at best a temporary remedy, opening the door for whichever is indicated, the relaxant or the constrictor, as may be indicated in each case—but it facilitates the action of either.

In the cases described by Lerch it would seem that ergotin might prove effective in full doses, such as would decisively contract the swollen gland, which is here a congeries of dilated vascular channels. This impression is confirmed by a case the writer noticed among Lerch's patients, a man whose face was covered with acne. It is well known that a full dose of ergotin will put an end to this eruption as long as the effects last, inducing a forcible contraction of the dilated tissues that leaves no trace of the disfigurement. The coincidence of acne points to the true pathologic state, and to the probability of ergotin acting similarly on both the engorged circulatory areas.

In the cases where there is failure of renal excretion, boldine seems to be the logical remedy, since it directly increases the formation and excretion of urea. Unfortunately, boldine is a remedy that is only useful or well tolerated in very small doses, for if these are increased beyond seven milligrams a day there arises a sense of uneasiness and apprehension that seems to point to some peril, and the patient finds immediate relief when the remedy is suspended, the unpleasant sensations returning if it is resumed in full doses. We learn to sense the presence of danger without actually seeing it.

In one such case I put the patient on a diet in which was a daily quart of buttermilk; and added, on account of a trace of bile in the urine, a scruple of sodium succinate daily. The urinary solids rose to three times their former point. Naturally, I credited this to the buttermilk; but by suspending each of the three remedies for periods it proved that each contributed to the result, by far the most potent being the succinate.

The paths of reflex irritation are many and complicated. It is well in neurotic maladies to leave no possibility neglected, and in every case of asthma we should give the patient the most scrupulously exact and comprehensive examination, detecting every aberration from normality and by appropriate treatment remedying it. We may not be able to conceive that a mole between the shoulders can possibly have anything to do with the malady—but remove the mole, nevertheless, and there may be an agreeable surprise in the way of immediate cessation of the paroxysms.

Here we may appreciate the value of the newer methods of diagnosis becoming available in late days. Examinations of the urine, feces and blood are advisable, besides the skilled physical investigations. Here, again, we are indebted to Dr. Lerch, for this accomplished New Orleans diagnostician has discovered in some of these asthmatics remarkable instances of splanchnoptosis. Displacement of the kidney, liver, stomach and intestine, coincident with asthma, should assuredly not be neglected or allowed to go without effective treatment.

Castro tabulates the exciting causes of asthma, premising the underlying abnormal excitability of the vagus:

A. Access of neuromotor origin:

1. Impression of organic powders as ipecacuanha.
2. Action of certain vapors.
3. Influence of the atmosphere.

B. Access of reflex origin:

1. Stomach and bowels.
2. Utero-ovarian organs.
3. Skin and sensory nerves.

C. Access of central origin:

1. Moral emotions.
2. Medullary lesions.

D. Access of humoral or mixed origin:

1. Alteration of the blood.
2. Empoisonments.
3. Vices of constitution.

Idiopathic asthma from a dynamic lesion of the pneumogastrics or of their bulbar center, is not as difficult of cure as is generally supposed. The spasmodic character of the malady imposes hyoscyamine as the dominant. With treatment methodic and sufficiently prolonged, the accesses lessen in frequency and intensity until they cease completely. Outside the access we should shun with the greatest care all the excitant causes capable of stimulating the pneumogastric, directly or indirectly. The nervous excitability is to be continuously combated by hyoscyamine (a milligram twice a day), increased progressively if after a month there is no evident result.

If there is also a diathesis, its modifiers should be added: For herpes acid arsenous, three milligrams a day; for arthrites colchicine, two milligrams a day; for all, the morning dose of laxative saline, and at bedtime the calmants of the grand systems, aconitine and digitalin, one or two milligrams of the former, twice this of the latter.

During the access, whatever the cause, the latent excitability of the vagus enters into action and manifests itself; whence results a loss of equilibrium in the nervous force, which concentrates itself especially in the muscles controlled by the pneumogastric, abandoning all the others, which are then tetanized. The well known phenomena then appear. Whenever there is a *dynamogenie* in one organic department, there is necessarily inhibition in another region connected more or less closely with the first. To these two lesions we oppose agents capable of ameliorating them, although they may appear antagonistic. We combat the spasm with hyoscyamine, one-half milligram every quarter hour; or we may use atropine or daturine. Hyoscyamine is least active, daturine most rapid, atropine lying between, and its salts being much less active than the pure alkaloid.

The paralysis is combated by morphine, alone or associated with strychnine, three milligrams of morphine and one-half milligram of strychnine for each one-half milligram of hyoscyamine. Hypodermics of morphine and atropine act with singular rapidity in affording relief.

When relief coincides with mucous secretion this should be activated by calcium sulphide three milligrams every half hour, or by iodoform in similar doses. These also soothe the nerves ending in the mucosa.

This treatment almost always gives good results. But if the physiologic effects are manifest before the therapeutic, and we are obliged to suspend the medication, we have recourse to camphor monobromide, three decigrams every quarter hour; or to lobelin, two milligrams every half hour. The latter directly calms the respiratory disturbance by its narcotic action, while by its local irritation it directs upon the stomach the accumulated nervous activity and thus indirectly calms the access.

During the height of the access the pulse hurries, the lung congests, often a fever develops, even to 104° F. Here one must not neglect digitalin to regulate the pulmonary circulation, adding aconitine when congestion is manifest—one-half milligram each every hour.

The main indication relates to the treatment during the intervals; to destroy the efficient cause of the malady. The directions must be followed rigorously to secure the object, and the habit of regular treatment confirms the cure. The fear of drugs induced by the old materia medica is our greatest obstacle.

The treatment may be thus tabulated:

I. Dominant, for pneumogastric hyperesthesia—Hyoscyamine.
II. Variant:
A. Causal:
Rheumatic diathesis—Colchicine, laxative saline.
Herpetic diathesis—Arsenous acid, laxative saline.
Congestion—Hemorrhoidal, aconitine, laxative saline.
Hepatic, etc.
Other influences—Suppress the cause.
B. Symptomatic:
Gastrointestinal aura—Lobelin.
Inspiratory spasm—Atropine, daturine, morphine.
Strychnine arsenate, camphor monobromide.
Catarrhal secretion—Calcium sulphide.
Iodoform.
Cardiac perturbation—Digitalin.
Congestive state—Aconitine.
Periodicity—Quinine hydrobromide.

Thierry-Mieg described the case of a man fifteen years asthmatic. He complained that he was always treated by emetics and antimony; leaving the stomach deranged for many days. He refused to endure such drugs and demanded something better. Strychnine and hyoscyamine were given, with a little kermes. Respiration improved but the throat became dry. Prompt relief was not secured. He was then given calcium sulphide three centigrams, iodoform one milligram, strychnine arsenate one-half milligram, every hour; codeine one milligram every two hours, hyoscyamine one-half milligram every four hours. Relief was so prompt and complete that the next visit, twenty-four hours later, was simply consultative. Treatment was continued two days, at longer intervals.

A restaurateur, obliged to go to the hall in an open vehicle every morning at 6, complained of respiratory oppression and commencing asthma. He was given calcium sulphide four centigrams as he entered the carriage and again as he left the hall. There was no more oppression, he expectorated a little, and found himself well.

A sea captain had suffered respiratory oppression for two years. He could take no precautions. Six centigrams of calcium sulphide daily were given him, and sufficed to remove the difficulty.

An old wine merchant had chronic bronchitis with profuse expectoration. He was quite meager. When he took twelve centigrams of calcium sulphide daily the sputa diminished one-half; he only awoke to spit once each night, and felt himself better.

These cases point to the possibility of the existence of an infectious element in asthma—often suspected but as yet not demonstrated. Still, we should not make the too common mistake of judging from the standpoint of our own perfect knowledge. The quest of the absolute is as far as ever from completion. The achievements of modern science pale to insignificance the imaginings of the Arabian Nights. In view of modern achievements, we are compelled to take refuge in the agnostic formula—we can not state that anything is or is not a possibility.

